# Association between dairy consumption and ischemic heart disease among Chinese adults: a prospective study in Qingdao

**DOI:** 10.1186/s12986-022-00645-9

**Published:** 2022-02-19

**Authors:** Jiahui Song, Chi Pan, Feifei Li, Yu Guo, Pei Pei, Xiaocao Tian, Shaojie Wang, Ruqin Gao, Zengchang Pang, Zhengming Chen, Liming Li

**Affiliations:** 1 Department of Nutrition and Food Hygiene, School of Public Health, Qingdao University, Qingdao, 266071 China; 2 Qingdao Municipality Center for Disease Control and Prevention, Qingdao, 266033 China; 3 Qingdao Institute of Preventive Medicine, Qingdao, 266033 China; 4 Chinese Academy of Medical Sciences, Beijing, 100730 China; 5 Clinical Trial Service Unit and Epidemiological Studies Unit (CTSU), Nuffield Department of Population Health, University of Oxford, Oxford, UK; 6 Department of Epidemiology and Biostatistics, School of Public Health, Peking University Health Science Center, Beijing, 100191 China

**Keywords:** China, Prospective study, Dairy, Ischemic heart disease

## Abstract

**Background:**

Previous studies linking dairy consumption with ischemic heart disease (IHD) are almost from western countries, with little from China. The present study was to explore the relationship between dairy consumption and IHD among Chinese adults.

**Methods:**

The data for the present study was from the prospective cohort study of China Kadoorie Biobank in Qingdao, a total of 33,355 participants in the present study. An interviewer-administered laptop-based questionnaire was used to collect information on the consumption frequency of dairy, incident IHD cases were identified through Disease Surveillance Point System and the new national health insurance databases. Cox regression analyses were performed to estimate adjusted hazard ratios (HRs) and confidence interval for the relationship between the incidence of IHD and dairy consumption.

**Results:**

The baseline survey reported that 32.4% of males and 34.6% of females consumed dairy regularly (i.e. ≥ 4 days/week). Over an average of 9.2 years follow-up, 2712 new-onset IHD were documented. Compared with participants who never or rarely consume dairy, the HR of consumed dairy regularly was 0.85(0.73–0.98) for males (*P* < 0.05), while no significant benefits were identified for females.

**Conclusions:**

Regular dairy consumption had an inverse association to the onset of IHD among males, with no similar findings for females.

**Supplementary Information:**

The online version contains supplementary material available at 10.1186/s12986-022-00645-9.

## Introduction

IHD is the leading cause of death worldwide [1]. The number of deaths caused by IHD reached 9.14 million and the number of disability-adjusted life years (DALYs) reached 182.03 million in 2019 [2]. In 2016, the IHD mortality rate ranked first, accounting for 40% of mortality in Chinese adults [3], indicating that it has become a serious public health issue [4]. IHD-related hospitalization rates annually increased 5.4% in the China Kadoorie Biobank (CKB) cohort study [5].

Dairy contains more saturated than unsaturated fat. Previous studies linked full-fat dairy consumption with an increased risk of IHD [6]. Some studies have suggested that dairy consumption has a positive or a neutral association with cardiovascular disease and all-cause mortality [7–9]. Observational studies from western countries suggested that the intake of full-fat dairy was in a neutral or inverse association with the onset of IHD [10, 11]. With higher consumption of butter and cheese, most Western countries' dietary guidelines recommended the intake of low-fat milk (i.e., milk fat < 0.5%) in place of full-fat dairy (i.e., milk fat > 3%) [12], while the WHO recommended an intake of whole milk or dairy products (e.g., cheese) of 250 g/day [13]. A study including 7354 healthy adults in Korea aged 40–69 supported that those who consumed higher amounts of milk or dairy products had a protective association with the cardiovascular system [14]. Another prospective cohort study conducted in China found that, compared with no consumption, increased consumption of dairy products had a lower risk of CVD mortality. Dairy consumption in China is much lower than in Western countries [15]. Consumption of dairy products continues to be low in China, with only a slight increase over time [16].

Existing evidence on the effect of dairy consumption on IHD is still controversial and mainly comes from Western countries. The association between dairy and IHD risk has rarely been investigated among Chinese adults. The present study investigated the relationship between dairy consumption and the risk of IHD.

## Materials and methods

### Study population

The participants of the present study came from the prospective survey of the China Kadoorie Biobank (CKB) in Qingdao. Details of the study have been previously reported [17–19]. A total of 35,508 residents, aged 30–79 years (born in 1930–1970) completed the baseline survey in 2004–2008. Participants who self-reported IHD (n = 1827), stroke (n = 238), or cancer (n = 162) at the baseline survey were excluded, and the final analysis included 33,355 participants.

The ethics board of the University of Oxford and the National, Shandong Provincial, and Qingdao Centers for Disease Control and Prevention in China all approved this study. All of the participants in the survey signed written informed consent forms.

### Data collection

The validated laptop-based questionnaire was completed by trained health workers, including sociodemographic information (age, education, occupation, household income, marital status), lifestyle (alcohol consumption, smoking status), family history, dairy products, and other diet frequency (rice, wheat, other staple foods, red meat, poultry, fish, eggs, fresh fruit, fresh vegetables, soybean, and preserved vegetables) in the last 12 months. The answer for the frequency of dairy (e.g., milk, yogurt) consumption included 5 groups (never/rarely, 1–3 d/month, 1–3 d/week, 4–6 d/week, daily). The questionnaire has good reproducibility and relative validity against multiple 24 h recalls (weighted κ was 0.78) [20].

Physiological measurements include body weight, height, waist circumference (WC), blood pressure, random glucose, etc. Body mass index (BMI) was calculated as weight (kg) divided by height squared (m^2^). Systolic blood pressure (SBP) and diastolic blood pressure (DBP): for each individual, blood pressure was measured twice and taken as the average. A third measure was required if the blood pressure difference was more than 10 mm Hg between the first two measures, and the average of the last two blood pressure values was recorded. Random blood glucose levels were measured immediately following sample collection using the SureStep Plus System (Johnson & Johnson, New Brunswick, NJ, USA).

### Follow-up for IHD and MCE

The primary outcome was the incidence and mortality of IHD (International Classification of Diseases-10: I20–I25) and MCE (major coronary event). MCE includes fatal IHD (I20–I25) and nonfatal myocardial infarction (I21–I23). This was ascertained through the Disease Surveillance Point System (DSPs) and the new national health insurance databases [21]. Participants were followed up from baseline until the date of IHD or MCE incidence or mortality, loss to follow-up, or December 31, 2015, whichever came first.

### Statistical analysis

The series of characteristics of the participants were described with frequency (N) and percentages (%) according to categories of dairy consumption, using Student’s t-test for continuous variables and the chi-square test for categorical variables.

To estimate the hazard ratios (HRs) and 95% confidence intervals (CIs) of dairy consumption and IHD risk, a Cox proportional hazards model was applied. The proportional hazards assumption for the Cox model was examined by testing the significance level of the interaction terms between dairy consumption and time. Potential confounding factors were adjusted in the different models. In Model 1, hazard ratios were adjusted for age (continuous variable) and sex (male or female); Model 2 was additionally adjusted for education (below high school, high school and above), occupation, marital status, household income (< 20,000 yuan or ≥ 20,000 yuan), diet frequency (egg, fresh vegetables, red meat, fresh fruit, poultry, soybean), smoking status (noncurrent smoking or current smoking), alcohol consumption (noncurrent drinking or current drinking), and metabolic equivalent of task (MET); Model 3 was additionally adjusted for BMI (continuous variable), SBP (continuous variable), DBP(continuous variable), random glucose (continuous variable), and family history of myocardial infarction (MI).

Subgroup analysis was performed to investigate the relationship between dairy consumption and the risk of IHD, according to baseline characteristics, including age (≤ 50 y or > 50 y), education (below high school, or high school and above), occupation (yes or no), marital status (yes or no), household income (< 20,000 yuan or ≥ 20,000 yuan), smoking status (noncurrent smoking or current smoking), alcohol consumption (noncurrent drinking or current drinking), MET (≤ 15 h/d or > 15 h/d), BMI (< 24 kg/m^2^, 24–28 kg/m^2^, and ≥ 28 kg/m^2^), SBP (< 140 mmHg or ≥ 140 mmHg), random glucose (< 11.1 mmol/L or ≥ 11.1 mmol/L), and family history (yes or no). The significance of the interaction was examined by the likelihood ratio test, comparing models with and without interaction terms between the stratifying variable and dairy consumption.

All *P* values were two-sided, and *P* < 0.05 was considered to be statistically significant. All analyses were performed using SPSS (version 25.0). All graphs were plotted using R 4.0.5 (https://www.R-project.org/).

## Results:

### Characteristics of the participants

Among the 33,355 participants, the mean (SD) age at baseline was 50.7 (10.0) years. A total of 32.4% of male and 34.6% of female participants consumed dairy daily (Table 1). Participants who consumed dairy more frequently were more likely to be female, and have a higher education level, a higher MET, lower blood pressure and lower BMI (*P* < 0.05) (Additional file 1: Table S1).

**Table 1:** Baseline characteristics of participants by frequency of dairy consumption

Characteristics	Dairy consumption					Overall
	Never	Monthly	1–3 day/week	4–6 day/week	Daily	
Number of participants	10,395	3555	6757	1446	11,202	33,355
Women (%)	55.6	52.3	53.7	56.6	56.8	55.3
Mean age (years)	51.8(9.6)	51.2(10.0)	49.5(9.6)	47.7(9.8)	50.6(10.5)	50.7(10.0)
High school and above (%)	26.1	35.8	41.2	49.8	41.6	36.4
Factory/Professional/sales (%)	50.4	55.4	61.2	69.8	58.7	56.7
Married (%)	92.0	92.5	94.6	94.9	92.7	92.9
Household income ≥ 20,000 (yuan) (%)	51.6	62.1	67.4	70.2	63.8	60.8
**Regular food consumption**^a^ (%)						
Eggs	47.5	43.2	41.2	40.0	66.0	51.7
Fresh fruit	47.5	41.3	50.4	50.1	67.3	54.2
Fresh vegetables	98.8	98.0	96.5	94.9	98.9	98.1
Soybean	6.8	4.9	4.6	6.4	9.9	7.2
Red meat	61.0	62.0	54.0	53.8	68.5	61.9
Poultry	1.3	1.7	3.9	2.1	1.9	2.1
Current drinking in men^b^ (%)	53.7	45.4	43.7	47.0	48.8	48.8
Current smoking in men^b^ (%)	65.6	58.6	54.2	54.1	57.2	59.2
MET (MET-hr/day)	15.2(10.1)	15.4(10.6)	17.0(10.9)	19.9(11.4)	17.6(10.1)	16.6(10.4)
BMI (Kg/m^2^)	26.3(3.7)	25.9(3.7)	25.5(3.4)	25.0(3.4)	25.4(3.6)	25.7(3.6)
SBP (mmHg)	132.3(22.5)	130.4(21.5)	128.9(21.5)	126.3(19.8)	128.2(20.9)	129.7(21.6)
DBP (mmHg)	78.9(10.9)	77.8(11.1)	77.6(10.8)	76.5(10.5)	77.6(10.5)	77.6(10.8)
Random glucose^c^ (mmol/L)	6.4(2.4)	6.4(2.9)	6.2(2.4)	6.0(2.1)	6.4(2.8)	6.3(2.6)
Family history of MI (%)	5.2	5.0	4.7	5.5	6.5	5.5

MET: exercise metabolic equivalent; BMI: Body Mass Index; SBP: Systolic Blood Pressure; DBP: Diastolic Blood Pressure; MI: myocardial infarction; Values are either percentage or mean (SD)

^a^Regular food consumption means consuming food products daily

^b^In women, only 1.5% current drinking and 1.0% current smoking

^c^422 participants had missing values for random glucose

### Association between dairy consumption and IHD

During an average of 9.2 years of follow-up (305,655.5 person-years), a total of 2712 incident IHD cases (1,075 men and 1637 women) and 420 incident MCE cases (271 men and 149 women) were documented (dditional file 1: Table S2), with an incidence of 887.3 (713.8 for men, and 892.0 for women) per 100,000 person-years. The multivariable Cox proportional hazards model was used to analyze the relationship between dairy consumption and the onset of IHD. After adjusting for major covariates, compared with participants who never or rarely consumed dairy, higher dairy intake had a neutral effect on the incidence and mortality of IHD and MCE (Table 2, 3, and dditional file 1: Table S3). The inverse association was stronger in men than in women, and no statistically significant association was found between dairy consumption and IHD in women.

**Table 2:** Risk of IHD, MCE and IHD mortality associated with consumption of dairy

Characteristics	Dairy consumption		
	Never	< 4 day/week	≥ 4 day/ week
**IHD**			
Cases	903	734	1075
PYs	92,292.98	88,778.41	114,812.61
Cases/PYs (/100,000)	978.41	826.78	936.31
Modle1	1.00	0.98(0.89–1.08)	0.93(0.85–1.01)
Modle2	1.00	0.97(0.88–1.07)	0.95(0.87–1.04)
Modle3	1.00	0.97(0.88–1.08)	0.99(0.90–1.09)
**MCE**			
Cases	142	108	170
PYs	95,075.64	90,932.75	118,238.13
Cases/PYs (/100,000)	149.35	118.77	143.78
Modle1	1.00	0.90(0.70–1.16)	0.89(0.70–1.11)
Modle2	1.00	0.90(0.70–1.17)	0.89(0.71–1.12)
Modle3	1.00	0.92(0.71–1.19)	0.94(0.74–1.18)
**IHD mortality**			
Cases	89	73	100
PYs	95,513.82	91,318.09	118,823.54
Cases/PYs (/100,000)	93.18	79.94	84.16
Modle1	1.00	1.04(0.76–1.42)	0.80(0.60–1.06)
Modle2	1.00	1.08(0.79–1.49)	0.84(0.63–1.13)
Modle3	1.00	1.08(0.78–1.48)	0.86(0.64–1.16)

For further statistical analysis, individuals of dairy product consumption were combined into three groups (never/rarely, < 4 days/week, ≥ 4 days/week)

Model 1: stratified by age-at-risk, gender (only in total population)

Model 2: as for model 1, additionally adjusted for education, occupation, marital status, household income, and food consumption (eggs, fresh fruit, fresh vegetables, soybean, red meat, poultry), alcohol consumption, smoking status, MET, family history of MI

Model 3: as for model 2, additionally adjusted for BMI, SBP, DBP, random glucose

**Table 3:** Risk of IHD associated with dairy among 14,908 male and 18,447 female participants

Characteristics	Dairy consumption		
	Never	< 4 day/week	≥ 4 day/ week
**Male**			
Cases	359	280	436
Modle1	1.00	0.88 (0.75–1.02)	0.87 (0.75–1.00)
Modle2	1.00	0.85 (0.73–1.00)	0.85 (0.73–0.98)
Modle3	1.00	0.84 (0.72–0.99)	0.86 (0.75–0.99)
**Female**			
Cases	544	454	639
Modle1	1.00	1.05 (0.93–1.19)	0.97 (0.86–1.08)
Modle2	1.00	1.07 (0.94–1.21)	1.04 (0.93–1.18)
Modle3	1.00	1.08 (0.95–1.23)	1.10 (0.97–1.24)

For further statistical analysis, individuals of dairy product consumption were combined into three groups (never/rarely, < 4 days/week, ≥ 4 days/week)

Model 1: stratified by age-at-risk, gender (only in total population)

Model 2: as for model 1, additionally adjusted for education, occupation, marital status, household income, and food consumption (eggs, fresh fruit, fresh vegetables, soybean, red meat, poultry), alcohol consumption, smoking status, MET, family history of MI

Model 3: as for model 2, additionally adjusted for BMI, SBP, DBP, random glucose

For men, dairy consumption < 4 days/week was inversely associated with the risk of IHD with an HR (95% CI) of 0.85 (0.73–1.00) and 0.85 (0.73–0.98) for dairy consumption ≥ 4 days/week (Model 2). Additionally adjusted for BMI, blood pressure, and random glucose, HR was attenuated to 0.84 (0.72–0.99) and 0.86 (0.75–0.99), respectively (Model 3).

### Subgroup analyses

**Fig. 1:**
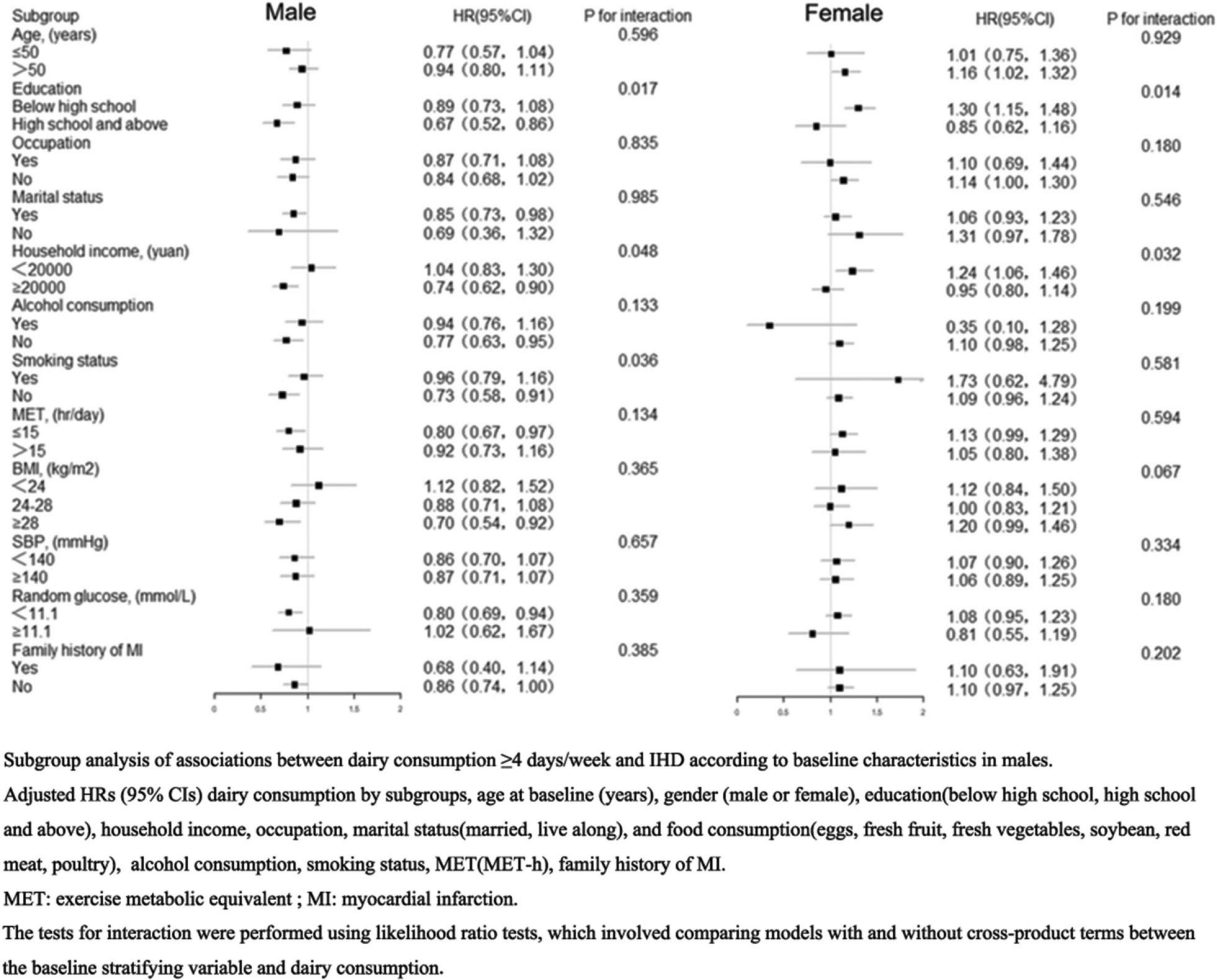
Subgroup analysis of associations between dairy consumption ≥ 4 days/week and IHD according to baseline characteristics in males. Adjusted HRs (95% CIs) dairy consumption by subgroups, age at baseline (years), gender (male or female), education(below high school, high school and above), household income, occupation, marital status(married, live along), and food consumption (eggs, fresh fruit, fresh vegetables, soybean, red meat, poultry), alcohol consumption, smoking status, MET (MET-h), family history of MI. MET: exercise metabolic equivalent; MI: myocardial infarction

Subgroup analysis was performed to investigate the relationship between dairy consumption and the risk of IHD, according to baseline characteristics (Fig. 1). The association between dairy and IHD differed by education level (*P*_*interaction*_: 0.017 for men and 0.014 for women) and household income (*P*_*interaction*_: 0.048 for men and 0.032 for women). The inverse associations were more pronounced in men who had a high education level (HR 0.67, 95% CI 0.52–0.86) than men who had a lower education level (HR 0.89, 95% CI 0.73–1.08) (*P*_*interaction*_: 0.017) and were more pronounced in men who had a household income ≥ 20,000 (HR 0.74, 95% CI 0.62–0.90) than men who had a household income < 20,000 (HR 1.04, 95% CI 0.83–1.30) (*P*_*interaction*_: 0.048).

However, a strong positive association was observed among women with a lower level of education (HR 1.30, 95% CI 1.15–1.48) compared to women with a higher level of education (HR 0.85, 95% CI 0.62–1.16) (*P*_*interaction*_: 0.014) and was more pronounced in women whose household income was < 20,000 (HR 1.24, 95% CI 1.06–1.46) than women whose household income was ≥ 20,000 (HR 0.95, 95% CI 0.80–1.14) (*P*_*interaction*_: 0.032).

Stronger inverse associations were also observed among men who had noncurrent smoking (HR 0.73, 95% CI 0.58–0.91) than men who had current smoking (HR 0.96, 95% CI 0.79–1.16) (*P*_*interaction*_: 0.036). No similar associations were observed across subgroups stratified according to age, occupation, marital status, alcohol consumption, MET, BMI, SBP or family history of MI (*P*_*interaction*_ > 0.05).

In addition, stronger positive associations were also observed among women with age > 50 y (*P*_*interaction*_: 0.929), although the interaction test was not statistically significant.

## Discussion

This large-scale prospective study showed that the regular intake of dairy products had a significant protective effect on the onset of IHD in men. However, no similar association was observed among women.

### Association between dairy consumption and IHD

In line with previous studies [22, 23], participants with a higher intake of dairy were more likely to have a lower BMI and lower blood pressure. Increasing evidence has shown that dairy products are associated with lower blood pressure and arterial stiffness [24].

Dairy products are rich in nutrients [25] (e.g. amino acids, calcium, vitamins), and can be an important nutrient-dense constituent of a healthy diet, an excellent food choice for healthy people of all age groups. Nutrient content and bioactive ingredients in dairy vary greatly, and their impact on health outcomes cannot be characterized fully by the presumed effect of one nutrient as a single biomarker [26]. There are approximately 400 different fatty acids in dairy products, especially in full-fat milk, which is the most complex of all-natural fats [27]. *The Chinese Dietary Guideline (2016)* recommended that adults consume 300 g of dairy per day (e.g., milk, yogurt) [28]. However, as a country with a high uptake of plant food-based diets and a generally low intake of dairy products, Chinese adults consume few dairy products (e.g., butter, cheese), and the most common type of dairy consumption is liquid milk [29]. It has been reported that China accounted for only 3.5% of the world’s total dairy production, which is much lower than the average of developing countries. The average intake of dairy products was 24.7 g/d in 2012 among Chinese adults [30], and that of elderly adults was 32.7 g/d, which reached a mere 1.21% of the Chinese dietary guideline recommended level (300 g/d). Only 12% of participants reported the consumption of dairy products in CKB [18].

The Guangzhou Biobank Cohort Study (GBCS) reported that full-fat dairy has a protective association with cardiovascular disease [31]. However, this study was cross-sectional, reverse causation is possible, and it did not adjust for potential confounding by other factors in the diet. Dairy and dairy consumption in Sweden is the highest worldwide. A cohort study in Sweden [7] showed that heptadecanoic acid (17:0) in dairy was associated with a lower risk of IHD, with a relative risk of 0.86 (0.78, 0.96).

The Isfahan Cohort Study (ICS) [32] included 5,432 participants aged ≥ 35 years who were recruited from January to September 2001. During a median of 10.9 years of follow-up, compared with never consuming dairy, participants with higher dairy intake had an inverse association with IHD, HR (95% CI) was 0.81 (0.65–0.99). The Multi-Ethnic Study of Atherosclerosis (MESA) [23], including 5,209 participants aged 45–84 years old at baseline, found that the saturated fatty acids (SFAs) of dairy were associated with a lower IHD risk. In the fully adjusted model, each 5-g/d increase in consumption was associated with a 21% lower risk of IHD, HR (95% CI) was 0.84 (0.71–0.99). Each 5-unit increase in the percentage of dairy SF was associated with a 38% lower risk of IHD, HR (95%CI) was 0.71 (0.52–0.98), consistent with the findings of the current study.

In the current study, the association was more pronounced in men than in women. A previous meta-analysis reported an inverse association between milk and IHD only in men (RR: 0.93; 95% CI: 0.87, 0.99), with a mean milk intake of 313 ml/d [33], in line with the present study. Furthermore, it has been reported that higher consumption of dairy was related to a lower risk of metabolic syndrome among men [34–36], which might reduce the risk of IHD [37].

In the subgroup analysis of dairy consumption, the relationship between dairy consumption and IHD risk appeared to be more pronounced in men with higher education, household income ≥ 20,000 yuan, and noncurrent smoking. High socioeconomic status (SES) has been associated with a healthier lifestyle. Participants with a higher SES tend to consume more dairy. Additionally, it has been reported that smoking exacerbates the effects of both total cholesterol (TC) and higher high-density lipoprotein (HDL) on IHD [38]. Smoking is also an important risk factor for central obesity and is associated with DNA methylation and oxidative stress [39, 40]. Heavy smoking tends to have multiple other health-related risk factors, weakening the protection of dairy products.

However, the positive association between dairy consumption and IHD was more pronounced in women with a lower education level (below high school) and household income < 20,000 yuan. Lower SES was associated with lower levels of milk consumption. With a lower intake of dairy, the association was about the same. The mean age at natural menopause was 48.6 years in CKB [41], and women aged > 50 years are usually at this stage. The menopausal transition is associated with significant hormonal changes. A previous study reported that women after natural menopause had a higher IHD risk [42]. This may be attributed to the body hormone (e.g., estrogen) levels of women modified after menopause [43], while higher education was associated with a later age at natural menopause [44]. One cross-sectional study [45] reported that estrogen was associated with lower serum cholesterol, lower very-low-density lipoprotein (LDL) cholesterol, and higher high-density lipoprotein (HDL) cholesterol, which may protect women against fatal IHD. In addition, the rate of depression is also higher among women after menopause.

### The possible mechanisms underlying the association

Although the association between dairy consumption and IHD is still controversial, there is existing evidence supporting that dairy products are beneficially associated with IHD. First, higher dairy food consumption was associated with a lower TC [46]. Some types of SFAs in dairy might increase the concentration of HDL-C, which could reverse the cholesterol transport pathways, inhibit LDL-C oxidation, and prevent the inflammatory process [47]. Second, the large amount of calcium in dairy is associated with a lower incidence of hypertension [48], which in turn reduces the risk of IHD. Third, dairy products protect against glucose-induced impairments in vascular function by limiting glucose-induced oxidative stress [49]. In addition, proteins from dairy products promote vascular function by improving nitric oxide bioavailability. Some studies have suggested that full-fat dairy might be a source of bioactive peptides [50, 51], which may protect the microvasculature from the detrimental effects of sodium. However, more studies are needed to determine the precise mechanisms.

### Strengths and limitations of this study

The present study had several strengths. First, the current study includes a prospective study design and a large number of community-dwelling adults. Second, every participant was followed up for a relatively long time, and detailed information on general health status was collected at baseline, which helped to adjust for a number of potential confounding factors on the relationship between dairy consumption and IHD onset among Chinese adults. A few limitations do need to be considered. The lifestyle was assessed only at baseline, and it might have changed during the long-term follow-up. This study was unable to evaluate long-term trends of dietary patterns and might not necessarily reflect dietary habits over the follow-up. The dietary questionnaire design was relatively simple, collecting consumption data for only some of the major food groups instead of individual food items. Hence, information on dairy product types was not collected. However, it has been reported that the average intake of dairy was 21.8 kg/y in Shandong Province, mainly milk (65.7%) and yogurt (26%) [52]. In addition, the food frequency was self-reported, which cannot eliminate recall bias.

## Conclusion

In summary, dairy consumption had a protective association with the onset of IHD for men, particularly among men with high-level education and noncurrent smoking. Dairy consumption had a positive association for women who were older and had lower education levels. Therefore, different populations should be given different dietary recommendations for dairy to prevent IHD.

## Supplementary Information

**Additional file 1** **Table S1:** Baseline characteristics of participants by dairy consumption. **Table S2:** IHD incident characteristics of participants according to age. **Table S3:** Risk of MCE and IHD mortality associated with consumption of dairy among 14,908 male and 18,447 female participants..

## Data Availability

Details of how to access China Kadoorie Biobank data and details of the data release schedule are available from www.ckbiobank.org/site/Data+Access.

## Abbreviations

CKB: China Kadoorie Biobank
IHD: Ischemic heart disease
MCE: Incident major coronary event
HR: Hazard ratio
CI: Confidence interval
DALYs: Disability-adjusted life years
WC: Waist circumference
DSPs: Disease surveillance point system
MET: Exercise metabolic equivalent
BMI: Body Mass Index
SBP: Systolic blood pressure
DBP: Diastolic blood pressure
MI: Myocardial infarction
PYs: Pearson years
KoGES: Korean Genome Epidemiology Study Cohort
TC: Total cholesterol
HDL: Higher high-density lipoprotein
SFAs: Saturated fatty acids
GBCS: Guangzhou Biobank Cohort Study
PURE: Prospective Urban Rural Epidemiology Study
ICS: Isfahan Cohort Study
MESA: Multi-Ethnic Study of Atherosclerosis
SES: Socioeconomic status
