# Supplementary material for: Association between dairy consumption and ischemic heart disease among Chinese adults: a prospective study in Qingdao
Source: Nutr Metab (Lond). 2022 Feb 19;19:11. doi: 10.1186/s12986-022-00645-9 (PMC8858533; doi:10.1186/s12986-022-00645-9)
Supplement: Supplementary file 1 — Additional file 1 Table S1: Baseline characteristics of participants by dairy consumption. Table S2: IHD incident characteristics of participants according to age. Table S3: Risk of MCE and IHD mortality associated with consumption of dairy among 14,908 male and 18,447 female participants.. [file 12986_2022_645_MOESM1_ESM.docx]

Supplementary table

Table S1 Baseline characteristics of participants by dairy consumption

Table S2 IHD incident characteristics of participants according to age

Table S3 Risk of MCE and IHD mortality associated with consumption of dairy among 14,908 male and 18,447 female participants

Table S1 Baseline characteristics of participants by dairy consumption

| Characteristics | Dairy consumption | | | all |
| --- | --- | --- | --- | --- |
|  | Never/rarely | <4 day/week | ≥4 day/week |  |
| Number of participants | 10395 | 10312 | 12648 | 33355 |
| Women (%) | 55.6 | 53.2 | 56.8 | 55.3 |
| Mean age (years) | 50.7±9.6 | 49.2±9.5 | 50.4±10.4 | 50.1±9.9 |
| High school and above (%) | 26.1 | 39.3 | 42.6 | 36.4 |
| Factory/Professional/sales (%) | 50.4 | 59.2 | 59.9 | 56.7 |
| Married (%) | 92.0 | 93.9 | 92.9 | 92.9 |
| Household income≥20,000 (yuan) (%) | 51.6 | 65.6 | 64.5 | 60.8 |
| Regular food Consumption^a^ (%) |  |  |  |  |
| Eggs | 47.5 | 41.9 | 63.0 | 51.7 |
| Fresh fruit | 47.5 | 47.3 | 65.3 | 54.2 |
| Fresh vegetables | 98.8 | 97.0 | 98.4 | 98.1 |
| Soybean | 11.4 | 9.5 | 17.0 | 12.9 |
| Red meat | 61.0 | 56.7 | 66.8 | 61.9 |
| Poultry | 27.9 | 39.2 | 40.8 | 36.3 |
| Current drinking in men^b^ (%) | 53.7 | 44.3 | 48.6 | 48.8 |
| Current smoking in men^b^ (%) | 65.6 | 55.7 | 56.9 | 59.2 |
| MET (MET-hr/day) | 17.7±11.5 | 18.9±11.8 | 19.0±1.1 | 18.6±11.5 |
| BMI (Kg/m^2^) | 25.9±3.6 | 25.5±3.4 | 25.3±3.4 | 25.6±3.4 |
| SBP (mmHg) | 133.3±21.5 | 130.9±20.5 | 130.1±22.2 | 131.3±20.7 |
| DBP (mmHg) | 80.4±11.5 | 79.4±11.3 | 78.1±11.0 | 79.2±11.3 |
| Random glucose^c^ (mmol/L) | 6.3±2.4 | 6.2±2.6 | 6.3±2.7 | 6.3±2.6 |
| Family history of MI (%) | 5.1 | 4.7 | 6.2 | 5.4 |

Individuals of dairy product consumption were combined into three groups (never/rarely, ＜4 days/week, ≥4 days/week).

MET: exercise metabolic equivalent; BMI: Body Mass Index; SBP: Systolic Blood Pressure; DBP: Diastolic blood pressure; MI: myocardial infarction; Values are either percentage or mean (SD)

^a^ Regular food consumption means ‘daily’ for eggs, fresh fruit, fresh vegetables, red meat; and ‘≥4 days/week’ for soybean, poultry

^b^ In women, only 1.5% current drinking and 1.0% current smoking

^c^ 422 participants had missing values for random glucose

Table S2 IHD incident characteristics of participants according to age

| Baseline age(y) | Incident age(y) | PYs | IHD incident | |
| --- | --- | --- | --- | --- |
|  |  |  | Cases | Cases/PYs(/100,000) |
| 30~ | 49.5±3.2 | 113289.5 | 236 | 208.3 |
| 45~ | 59.0±3.1 | 110338.7 | 865 | 784.0 |
| 55~ | 67.7±3.4 | 52303.6 | 860 | 1644.2 |
| 65~79 | 77.3±3.7 | 29723.7 | 751 | 2526.6 |
| All | 63.6±9.2 | 305655.5 | 2712 | 887.3 |

HR: indicates hazard ratio; CI, confidence interval; PYs: Pearson years

Table S3Risk of MCE and IHD mortality associated with consumption of dairy among 14,908 male and 18,447 female participants

| Characteristics | Dairy consumption | | |
| --- | --- | --- | --- |
|  | Never | ＜4 day/week | ≥4 day/ week |
| **MCE** |  |  |  |
| **male** |  |  |  |
| Cases | 93 | 74 | 104 |
| Model1 | 1.00 | 0.89(0.66-1.21) | 0.80(0.61-1.06) |
| Model2 | 1.00 | 0.88(0.64-1.21) | 0.81(0.60-1.08) |
| Model3 | 1.00 | 0.89(0.65-1.22) | 0.85(0.63-1.13) |
| **female** |  |  |  |
| Cases | 49 | 34 | 66 |
| Model1 | 1.00 | 0.91(0.59-1.41) | 1.09(0.75-1.57) |
| Model2 | 1.00 | 0.93(0.59-1.45) | 1.09(0.75-1.61) |
| Model3 | 1.00 | 0.95(0.60-1.49) | 1.19(0.80-1.75) |
| **IHD mortality** |  |  |  |
| **male** |  |  |  |
| Cases | 54 | 42 | 56 |
| Model1 | 1.00 | 0.91(0.61-1.36) | 0.71(0.49-1.03) |
| Model2 | 1.00 | 0.98(0.65-1.49) | 0.75(0.51-1.10) |
| Model3 | 1.00 | 0.97(0.64-1.48) | 0.76(0.51-1.12) |
| **female** |  |  |  |
| Cases | 35 | 31 | 44 |
| Model1 | 1.00 | 1.23(0.76-2.00) | 0.87(0.61-1.49) |
| Model2 | 1.00 | 1.18(0.72-1.94) | 0.98(0.62-1.54) |
| Model3 | 1.00 | 1.19(0.72-1.97) | 1.04(0.65-1.64) |
| **MCEmortality** |  |  |  |
| **male** |  |  |  |
| Cases | 54 | 42 | 56 |
| Model1 | 1.00 | 0.91(0.61-1.36) | 0.71(0.49-1.03) |
| Model2 | 1.00 | 1.00(0.66-1.52) | 0.77(0.52-1.13) |
| Model3 | 1.00 | 0.98(0.65-1.49) | 0.75(0.51-1.11) |
| **female** |  |  |  |
| Cases | 35 | 31 | 44 |
| Model1 | 1.00 | 1.23(0.76-2.00) | 0.95(0.61-1.49) |
| Model2 | 1.00 | 1.17(0.71-1.92) | 1.00(0.63-1.58) |
| Model3 | 1.00 | 1.17(0.71-1.94) | 1.03(0.65-1.64) |

For further statistical analysis, individuals of dairy product consumption were combined into three groups (never/rarely, ＜4 days/week, ≥4 days/week).

Model 1: stratified by age-at-risk, gender (only in total population)

Model 2: as for model 1, additionally adjusted for education, occupation, marital status, household income, and food consumption (eggs, fresh fruit, fresh vegetables, soybean, red meat, poultry), alcohol consumption, smoking status, MET, family history of MI

Model 3: as for model 2, additionally adjusted for BMI, SBP, DBP, random glucose
